# Recycled PET Sand for Cementitious Mortar

**DOI:** 10.3390/ma15010273

**Published:** 2021-12-30

**Authors:** Angélica Faria Campanhão, Markssuel Teixeira Marvila, Afonso R. G. de Azevedo, Tulane Rodrigues da Silva, Roman Fediuk, Nikolai Vatin

**Affiliations:** 1 LAMAV-Advanced Materials Laboratory, State University of the Northern Rio de Janeiro, Av. Alberto Lamego, 2000, Campos dos Goytacazes 28013-602, Brazil; angelicacampanhao@gmail.com (A.F.C.); afonso@uenf.br (A.R.G.d.A.); tuhrodrigues_@hotmail.com (T.R.d.S.); 2 CRP–Rio Paranaíba Campus, UFV-Federal University of Viçosa, Rodovia BR 230 KM 7, Rio Paranaiba 38810-000, Brazil; 3 LECIV-Civil Engineering Laboratory, State University of the Northern Rio de Janeiro, Av. Alberto Lamego, 2000, Campos dos Goytacazes 28013-602, Brazil; 4 Polytechnic Institute, Far Eastern Federal University, 690922 Vladivostok, Russia; 5 Peter the Great St. Petersburg Polytechnic University, 195251 St. Petersburg, Russia; vatin@mail.ru

**Keywords:** PET, recycling, mortars, Portland cement

## Abstract

Cementitious materials cause a great impact on the environment due to the calcination of clinker and the extraction of non-renewable mineral resources. In this work, the replacement of quartz sand from the river by PET sand was evaluated at levels of 10%, 20%, and 30%. Tests were performed in the fresh state through consistency, air retention, density, and incorporated air and in the hardened state for compressive strength, flexural strength, density, capillarity, and water absorption. The results show that PET sand is viable in contents of up to 10%, improving the mechanical properties of the mortar and without compromising its workability and incorporated air properties. Above that level, the loss of properties is very excessive, mainly of workability and incorporated air. The incorporated air of the 30% composition, for example, reaches 24%, an excessive value that impacts the properties of the hardened state, making it impossible to use the material at levels greater than 20%. It is concluded that the use of recycled PET sand is a possibility that contributes to sustainable development, as it reduces the extraction of quartz sand from the river, a non-renewable mineral resource.

## 1. Introduction

Mortars are construction materials used for different purposes in civil construction, such as laying blocks, covering ceilings and walls, or smoothing surfaces [1,2]. They are usually produced with binders, such as Portland cement and hydrated lime, fine aggregate, and water. The use of these materials is problematic from an environmental point of view, mainly due to the clinkerization needed to obtain Portland cement and the high extraction of mineral resources to produce the binders and to be used as fine aggregate [3,4,5].

Quartz sand from rivers or dunes is usually used as fine aggregate in concrete and mortar. However, the extraction of this material is highly harmful to the environment due to the lack of natural recovery of mineral resources, which are extracted at a much faster rate than they are formed [6,7]. This situation demands the attention of researchers from all over the world, who started to develop research aiming to use recycled residues as aggregates in cementitious materials.

Some relevant research that evaluated the use of recycled materials replacing natural sand are highlighted as follows: Azevedo et al. (2020) [8] evaluated the replacement of natural sand by construction and demolition waste at levels of 25%, 50%, and 100%. There was a gain in mechanical properties with the use of RCD, associated with the better packing provided by the residue. It is known that the material used as sand must be inert and must not interfere with the hydration of the cement and the reaction of other binders. However, as shown by [8,9], this material must present adequate particle size to reduce porosity, contributing to the packing and mechanical strength of cementitious materials. The results obtained by Azevedo et al. (2020) [8] were promising and justify the objective of the present research.

Gencel et al. (2021) [6] evaluated the replacement of natural sand by expanded vermiculite in geopolymeric mortars. The authors evaluated 15% and 30% replacement contents and found that, in addition to the reduction in density, which was already expected due to the nature of the expanded vermiculite, the mechanical strength results were equivalent to the reference composition produced with natural sand. In addition, there were positive results in the thermal conductivity tests, proving the feasibility of replacing natural sand. Other promising research has also evaluated the replacement of natural sand: Marvila et al. (2020) [10], who evaluated the replacement of natural sand by rock residue in gypsum mortars for restoration of historic structures and Amaral et al. (2020) [11], who evaluated the replacement of natural sand by rock residue in cement mortars for wall cladding and block laying, among other researches that verified the use of paper waste [12,13,14] and glass waste [15,16] as sand in cementitious mortars.

In all these researches, the successful replacement of natural sand is only possible when the recycled material has an adequate granulometry, favoring packing, and when the material is inert. This feature is a great attraction for the use of PET waste since the material is inert in cementitious materials and favors packaging when properly used. This was tested in the research by Silva et al. (2021) [17], where the authors used PET waste to produce soil cement blocks. The results obtained by the authors prove that the material does not chemically interact with the cement but allows an increase in mechanical strength. This proves the reduction in porosity of this type of recycled aggregate.

In addition to its use in cementitious materials, it is observed that PET has applications when used to reinforce polymer composites. Mhanna et al. (2020) [18] evaluated how the thermal effect alters the mechanical properties of fiber-reinforced polymer (FRP) systems with PET through numerical modeling and experimental research. The authors concluded that FRPs produced with PET have a different behavior from traditional materials, where high levels of ductility of these materials are observed before failure in comparison with conventional FRPs. This is a positive feature that demonstrates the advantages of using PET in different types of materials.

In addition to the direct application of PET, other works highlight the application of polymeric systems reinforcing cementitious materials, such as the work by Hawileh et al. (2014) [19], where the authors evaluated the mechanical behavior of concrete beams reinforced with polymeric systems. The results obtained highlight that the use of polymeric systems increases the ductility of concrete beams, a beneficial factor since cementitious materials present problems regarding this property.

These results were confirmed in other later studies, such as the works by Hawileh et al. (2019) [20] and Naser et al. (2021) [21], where through experimental research, modeling using Finite element modeling, and through bibliographic consultation, the authors proved the use of polymeric materials (such as PET) together with cementitious materials. In addition, they proved the ductility gain that these materials provide when applied in conjunction with concrete and other cement-based materials.

Considering this information, it is noteworthy that the objective of this manuscript is to evaluate the influence of recycled PET sand as a substitute for natural sand at levels of 0–30% in cementitious mortars. The main properties in the fresh state were evaluated, such as consistency index, incorporated air, and water retention, and the main properties in the hardened state through tests of tensile strength in bending, compressive strength, density, and water absorption.

## 2. Materials and Methods

The materials used in the research were ordinary Portland cement (OPC) composite, containing approximately 80% clinker and 20% blast furnace slag, and hydrated lime (CH), containing at least 90% hydrated composite. In addition, natural sand from a quartz river, extracted in Campos dos Goytacazes, RJ, Brazil, was used. In addition, recycled PET sand extracted from an industry in the region was used, with the particle size adjusted for applications in cementitious materials.

**Figure 1:**
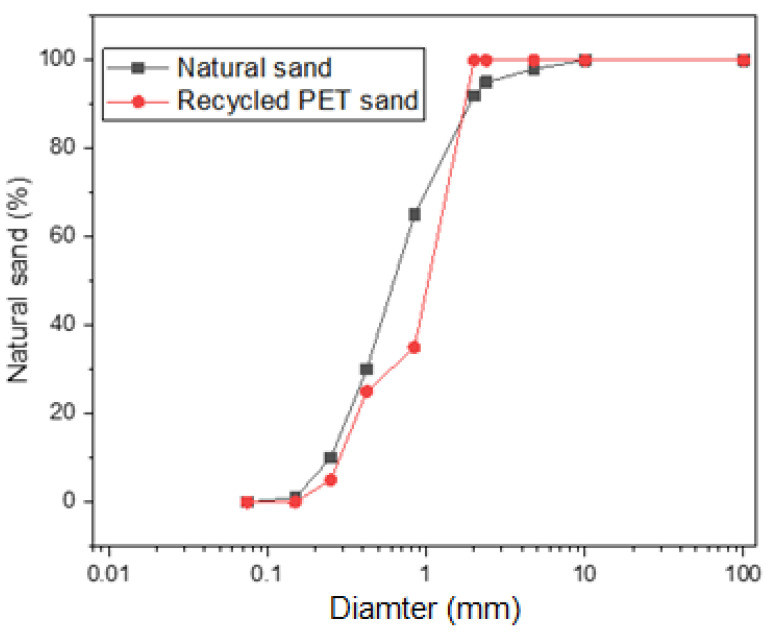
Granulometry of aggregates.

Figure 1 shows the granulometry of the aggregates used. It is important to highlight that the granulometry of recycled PET sand was chosen to be equivalent to the granulometry of natural sand. This can be proven by the curvature coefficient of the aggregates, which was 3.0 for natural sand and 2.9 for recycled sand, and by the uniformity coefficient, calculated as 0.9 for natural aggregate and 1.1 for the recycled aggregate.

The composition of mortar used in the research was 1:1:6:1.4 (cement: hydrated lime: sand: water), chosen based on previous works that evaluated mortars for application in wall coverings and for settlement of blocks [1,3,22,23]. The replacement performed was in levels from 0 to 30%, using the amounts defined in Table 1.

The quantities established in Table 1 were mixed in a mortar, following the procedures of NBR 13276 [24] and EN-BS 4551 [25]. They were then used to assess the fresh-state tests. The tests carried out were the consistency test, which consists of measuring the horizontal spread of the mortar under controlled conditions, correlating this information with the workability of the material. Still in the fresh state, mass density and incorporated air tests were carried out by the pressureometric method, following NBR 13278 [26] and water retention by the modified Buchner funnel method, using the recommendations of NBR 13277 [27] and EN-BS 4551 [25].

Then, 40 × 40 × 160-mm prismatic specimens were molded, always using 3 repetitions for each composition and in each test evaluated in the hardened state. The molding of the samples followed the procedure of NBR 13279 [28]. The curing procedure was carried out at an ambient temperature of 25 °C, for 28 days, according to NBR 13279 [28] and EN-BS 4551 [25]. This same standard sets the standards for flexural strength and compressive strength tests, carried out with the aid of an Instron universal test press with a 1000-kg capacity S charge bill. The speed used in the test was 50 N/s in the flexural strength test and 500 N/s in the compressive strength test.

Other tests were carried out in a complementary way in the hardened state: mass density test, through NBR 13280 [29] and water absorption test by immersion, using the procedures of NBR 9778 [30]. Finally, the capillary test was carried out to assess the capillary phenomena in the material, using the procedure of NBR 15259 [31] and EN-BS 4551 [25].

## 3. Results and Discussion

**Figure 2:**
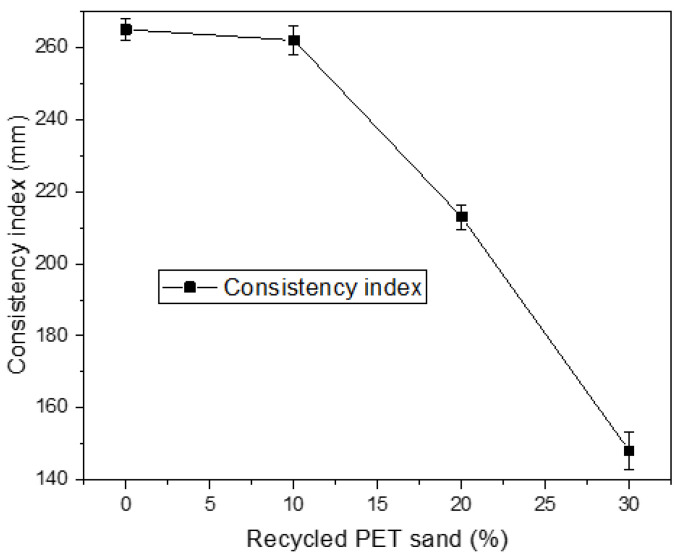
Consistency Index Results.

Figure 2 presents the consistency index results. It is observed that the composition of 0% and 10% present results compatible with the international bibliography, which recommends the use of a limit of 265 to 255 mm of scattering [23,32]. However, the 20% and 30% compositions showed a high loss of workability. This is related to the absorption of water promoted by recycled PET sand, which sequesters the free water available to improve spreading, and consequently increases the internal friction of the grains that make up the mortar, excessively impairing workability [33]. This characteristic even harms other properties of the material in the hardened state, such as mechanical resistance and water absorption, since the lack of workability prevents the molding of specimens from taking place efficiently.

**Figure 3:**
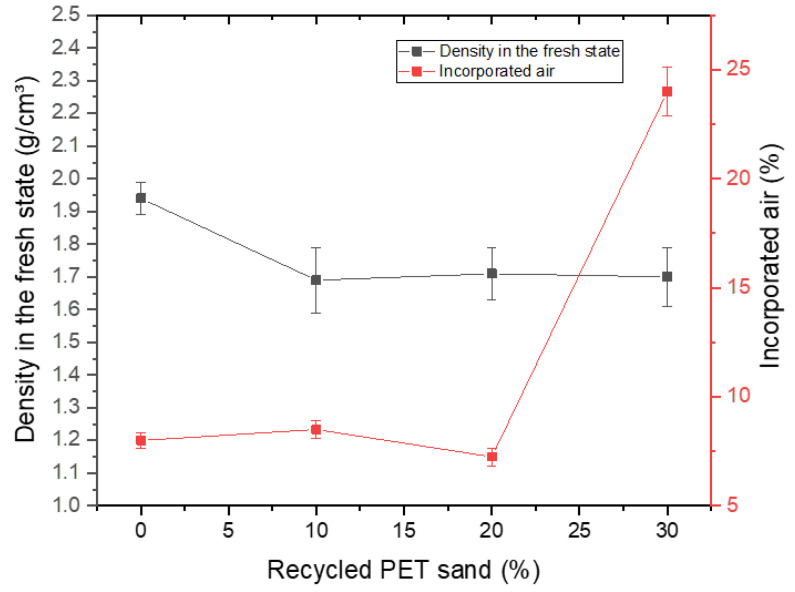
Density results in fresh state and incorporated air.

Figure 3 shows the density results in the fresh state and incorporated air. A reduction in density in the fresh state is observed with the use of recycled PET sand. This can be explained by the density of the material, approximately 1.65 g/cm³, while the density of natural sand is 2.65 g/cm³. Density reduction is something positive as long as it does not affect the other properties studied [11,34]. The incorporated air content, on the other hand, has an adverse behavior. It is observed that compositions 0%, 10%, and 20% do not have similar incorporated air, around 7.5%. However, the 30% composition has a very excessive incorporated air content, around 24%. This is a big problem, as it causes porosity in the hardened cementitious matrix and reduces mechanical strength. It can be seen in the figure that there was a large increase in incorporated air, as the PET incorporation content increased from 20% to 30%. This change cannot be attributed to the difference in density, since, as seen in Figure 3, there was not the same change in behavior in this property. Thus, the best explanation for the increase of air incorporated in the composition 30% is related to the lack of homogeneity of the material and the absorption of water from recycled PET sand, which forms a surface with low adhesion to cement in the areas surrounding the material, responsible by lifting the incorporated air [35].

**Figure 4:**
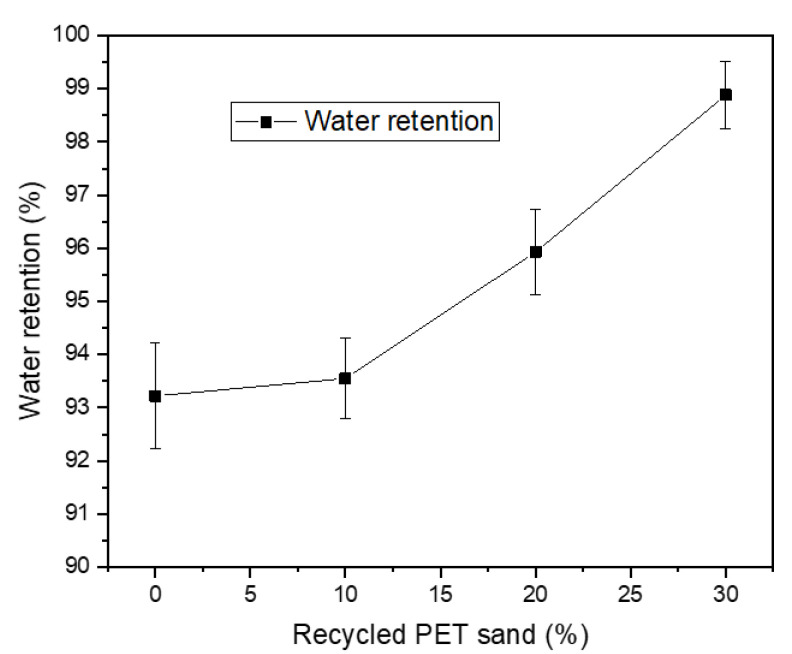
Water retention results.

Figure 4 presents the water retention results. An increase in water retention is observed as the recycled PET sand content increases, which is directly related to the material’s water absorption. The water retention of mortars must not present values below 75%, as this can harm the mechanical resistance of the material developed through the hydration of the OPC [12]. However, the retention cannot be higher than 95% because in this case, there will be a deficiency in the adhesion between the mortar and the applied substrate [36]. This adhesion occurs through stress transfer bridges developed when the substrate can suck cement paste from the mortar. In this sense, the 20% and 30% mortars do not meet the requirements established by international references.

**Figure 5:**
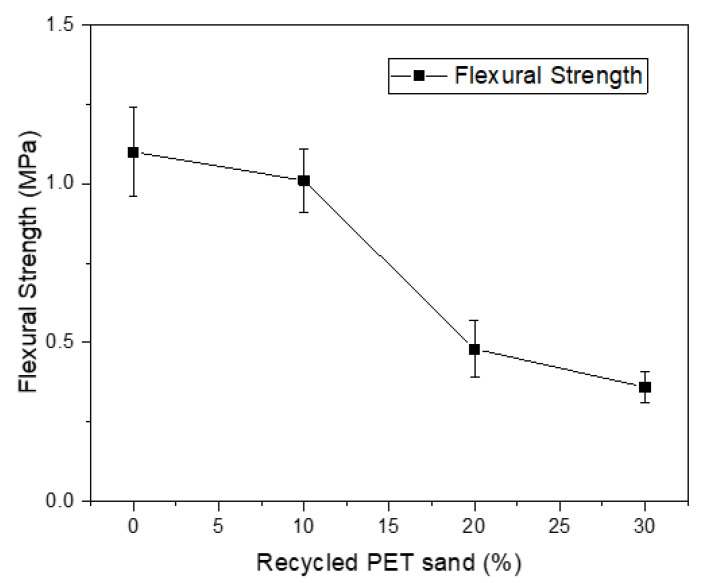
Flexural strength results.

**Figure 6:**
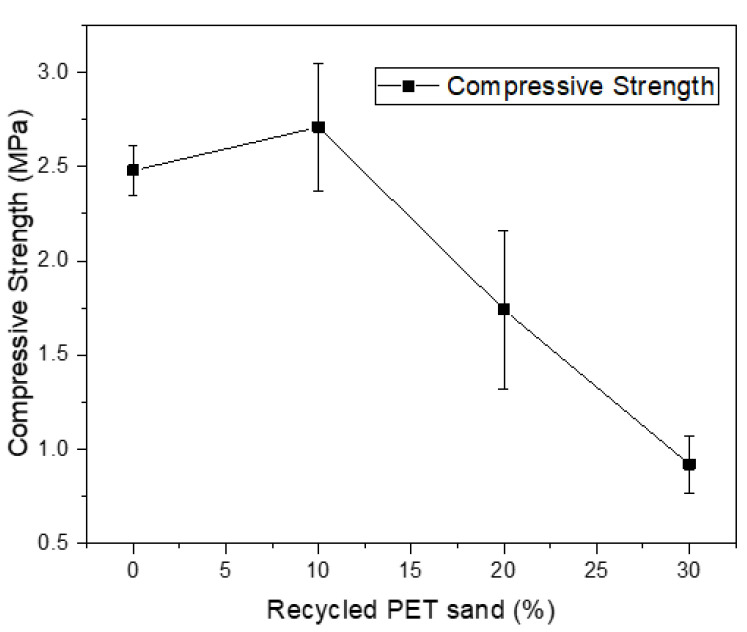
Compressive strength results.

Figure 5 presents the results of flexural strength of mortars, while Figure 6 presents the results of compressive strength. According to authors who study this type of material, the flexural strength of mortars varies between 1 and 4.5 MPa. Some authors even suggest that the mortar cannot present excessive strength values because this makes the material more rigid, compromising the performance of the material when it is in service [1]. Azevedo et al. (2020) [12] obtained values ranging from 0.8 MPa to 1.1 MPa replacing natural sand with sand from waste paper; Marvila et al. (2019) [3] obtained values from 1.0 MPa to 1.1 MPa with the same mortar composition. Similar results were obtained by Bonfim et al. (2021) [37] and Souza et al. (2020) [38]. Therefore, the flexural strength values are compatible. However, mortars with 20% and 30% recycled PET sand are less than 1 MPa and are not compatible with the application. An interesting way to reduce the drop in strength would be the use of PET in its saturated form. This would reduce the water absorption caused by using PET in dry form and would likely have a positive impact on the mechanical properties, which would not have such a sharp drop, as seen in Figure 5 and Figure 6.

Compressive strength, on the other hand, usually presents higher values since cementitious materials are very resistant to compression. Some results obtained by other authors are highlighted as follows: Azevedo et al. (2020) [8] obtained values of approximately 2.63 MPa, while Azevedo et al.’s (2020) [12] values were around 2.6 MPa. The results obtained for the 0% and 10% compositions are compatible, but the values for the 20% and 30% compositions are very low and are not possible for the proposed application. It is observed that the 30% composition showed a very sharp drop in strength. This is directly related to the high content of air incorporated in the material, which influenced the porosity of the material and consequently provided the reduction of mechanical properties.

**Figure 7:**
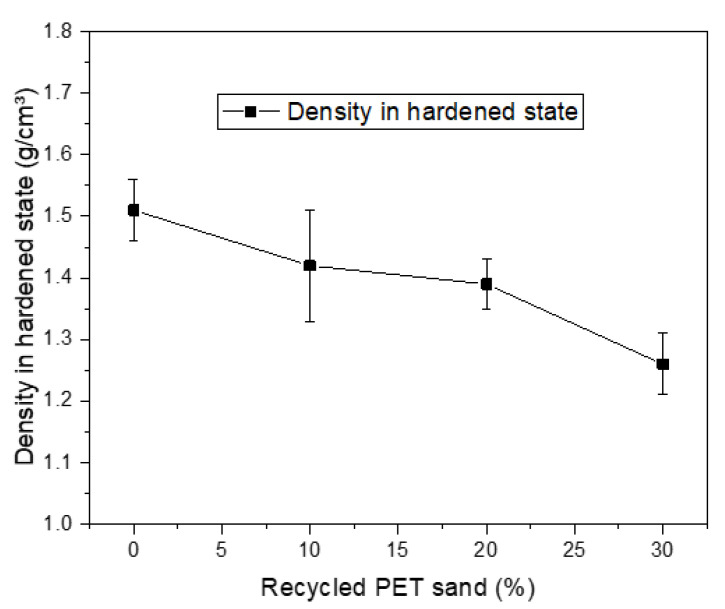
Density results in the hardened state.

Figure 7 shows the mass density results in the hardened state. Again, a reduction in density is observed as larger amounts of recycled PET sand are used. This feature is beneficial if no other factors are harmed. The values obtained are compatible with other similar studies, such as by Bonfim et al. (2021) [37] and Souza et al. (2020) [38].

**Figure 8:**
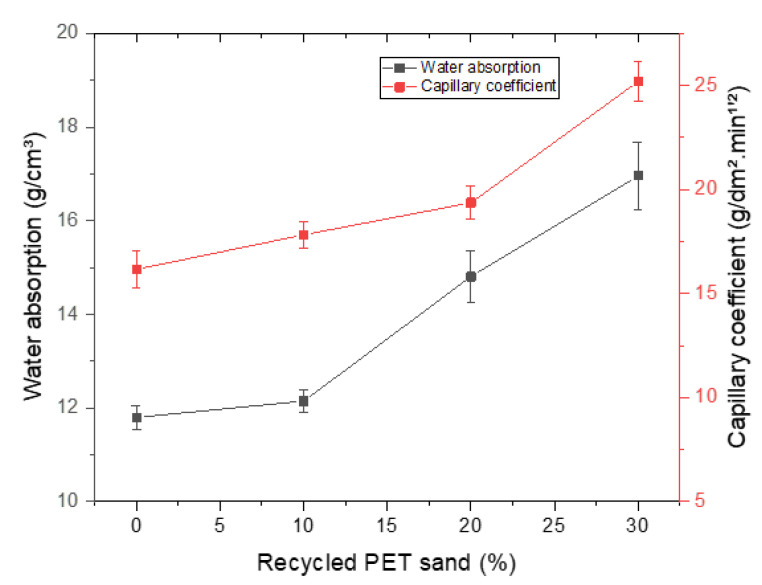
Water absorption and capillary results.

Figure 8 presents the results of water absorption and capillarity. It is observed that water absorption increases with increasing PET recycled sand content. The mortars containing 20% and 30% of PET again present excessive values, above 14%. Comparing the results of other researches, it is observed that Souza et al. (2020) [38] obtained values between 15 and 16.8%, while Marvila et al. (2020) [39] obtained values between 12 and 14%. Thus, it is observed that the values obtained for water absorption are compatible although the 20% and 30% compositions have presented excessive values. This may be associated with an increase in the porosity of the material and, consequently, a decrease in mechanical strength, observed in Figure 5 and Figure 6.

The capillary results are related to the phenomenon of capillary water absorption and are problematic in mortars. It is observed that 0%, 10%, and 20% had capillarity coefficient below 20 g/dm².min^1/2^; however, the 30% composition again presented excessive and non-standard values. Comparing with the results of other researches, it is observed that Azevedo et al. (2020) [8] resented a capillary coefficient between 15 and 18 g/dm².min^1/2^; Bonfim et al. (2021) [37] obtained values around 20 g/dm².min^1/2^; and Azevedo et al. (2020) [12] presented values ranging between 14 and 18 g/dm².min^1/2^. Therefore, the results obtained are consistent.

## 4. Conclusions

Based on the results obtained, it is possible to conclude that:

- The use of recycled PET sand in contents of 20% and 30% promotes a drop in the consistency index and a drop in workability due to the water absorption of the PET. These compositions, especially the one with 30%, presented an excess of incorporated air and low values of flexural and compressive strength when compared to other mortars and other similar researches. In addition, the use of 20% and 30% contents promoted an increase in porosity, as observed in the results of water absorption and capillarity.
- The composition with 10% recycled PET sand did not harm the properties of the mortar, presenting values very close and equivalent to the reference composition containing only natural sand. These results prove the viability of using recycled PET sand at 10% levels in cement mortars.

## Figures and Tables

**Table 1:** Compositions studied in the research.

Composition	OPC (g)	CH (g)	Natural Sand (g)	Recycled PET Sand (g)	Water (g)
0%	150	150	900	0	210
10%	150	150	810	90	210
20%	150	150	720	180	210
30%	150	150	630	270	210

## Data Availability

Not applicable.
